# A Case of Serotonin Syndrome Associated With Acute Quetiapine Poisoning

**DOI:** 10.1155/crcc/5831415

**Published:** 2026-05-19

**Authors:** Dan Wu, Haidong Qin

**Affiliations:** ^1^ Department of Emergency Medicine, Nanjing First Hospital, Nanjing Medical University, Nanjing, China, njmu.edu.cn

**Keywords:** poisoning, quetiapine, serotonin syndrome

## Abstract

A 21‐year‐old woman was admitted to our emergency department following a 3‐h loss of consciousness after intentional ingestion of multiple psychotropic medications. Initial management included gastric lavage, catharsis, intravenous fluid resuscitation, diuresis, and hemoperfusion to enhance toxin elimination. Shortly after admission, she developed rapid respiratory deterioration, high fever, limb tremors, and tachycardia. Emergency endotracheal intubation and mechanical ventilation were initiated, along with sedation and active cooling measures. A comprehensive workup—including complete blood count, procalcitonin (PCT), blood, urine, and sputum cultures, and chest CT—was performed to exclude infectious etiologies. After ruling out other potential causes, a diagnosis of serotonin syndrome was related to quetiapine fumarate overdose. With aggressive supportive care and enhanced drug clearance, the patient gradually regained consciousness, achieved hemodynamic stability, and was successfully weaned from mechanical ventilation before being discharged without neurological sequelae. Clinicians should maintain a high index of suspicion for serotonin syndrome in patients with quetiapine overdose who present with altered mental status, autonomic instability, hyperthermia, and neuromuscular abnormalities. Early recognition and prompt symptomatic intervention are critical to improving clinical outcomes.

## 1. Introduction

Serotonin syndrome (SS) is a potentially life‐threatening clinical disorder characterized by excessive serotonergic activity in the central and peripheral nervous systems, resulting from the use of serotonergic agents alone or in combination. With the increasing clinical use of antidepressants, analgesics, and antiemetics, the incidence of SS has risen steadily, making it an increasingly recognized and critical concern in clinical practice. In 1982, Insel et al. [1] formally introduced the term “serotonin syndrome” based on convergent evidence from animal models and human clinical observations. Subsequently, in 1991, Sternbach et al. [2] described cases of hyperthermia, altered mental status, and muscle rigidity occurring during combined treatment with monoamine oxidase inhibitors (MAOIs) and tricyclic antidepressants, proposing the concept of “serotonin toxicity.” Since then, related terms such as “5‐HT toxicity,” “5‐HT storm,” or “5‐HT crisis” have been used interchangeably in the literature to describe this condition [3]. The clinical presentation of SS is highly variable: mild cases may present with tremor, diaphoresis, and diarrhea, whereas severe cases can rapidly progress to sustained hyperthermia, autonomic instability, and rhabdomyolysis, with mortality rates estimated between 2% and 12% [4]. The underlying pathophysiology primarily involves drug‐induced disruption of 5‐HT metabolism—through inhibition of reuptake, enhanced release, or impaired degradation—mediated via effects on transporters or metabolic enzymes, consistent with an acute toxicological mechanism. Reflecting this paradigm shift, the 2022 guidelines from the International College of Neuropsychopharmacology (CINP) have formally integrated “serotonin toxicity” into the broader diagnostic framework of “serotonin syndrome.” Most cases arise from the concomitant use of two or more serotonergic agents; monotherapy‐induced SS is rare but increasingly documented. Currently, no specific biomarker or laboratory test confirms the diagnosis. Clinical assessment relies on a detailed medication history, characteristic symptom clusters, and application of standardized diagnostic criteria such as the Hunter or Sternbach criteria. Nevertheless, differential diagnosis remains challenging due to overlapping features with neuroleptic malignant syndrome (NMS), anticholinergic toxicity, and systemic infections. This report presents a case of typical SS following acute quetiapine overdose, systematically describing its clinical features, laboratory findings, and therapeutic management, along with a review of current literature. The aim is to enhance clinicians′ recognition of early warning signs, support rational prescribing practices, and inform individualized treatment strategies.

## 2. Patient′s Presentation

A 21‐year‐old female with a documented history of depression was admitted to our emergency department at 16:24 on April 4, 2025, presenting with loss of consciousness 3 h after intentional ingestion of multiple psychotropic agents. According to family members, the patient had self‐administered an unknown quantity of alprazolam, lorazepam, and quetiapine fumarate tablets approximately 3 h prior to presentation. Shortly thereafter, she developed confusion and vomiting. Upon arrival, she underwent immediate gastric lavage and intravenous fluid resuscitation; however, her neurological status progressively deteriorated, culminating in deep coma, necessitating transfer to the emergency intensive care unit (EICU) for advanced supportive care.

Initial physical examination revealed a temperature of 36°C, heart rate of 101 beats/min, respiratory rate of 25 breaths/min, blood pressure of 123/77 mmHg, and oxygen saturation of 96% on supplemental oxygen. Neurological assessment demonstrated a Glasgow Coma Scale (GCS) score of 3 (M1V1E1), consistent with profound central nervous system depression. Pupils were bilaterally equal and small (approximately 2 mm), with sluggish pupillary light reflexes. No rash, petechiae, or jaundice was observed. Pulmonary auscultation revealed coarse breath sounds with scattered moist rales; cardiac examination showed a regular rhythm without murmurs. The abdomen was flat, soft, nontender, and without rebound tenderness. Limb muscle strength was Grade 0, with reduced muscle tone. Pathological reflexes were absent, and meningeal signs were negative.

Laboratory investigations showed leukopenia (white blood cell count: 3.33 × 10^9^/L) with a neutrophil proportion of 49.9%; red blood cell and platelet counts were within normal limits. Comprehensive metabolic profiling—including liver and renal function, myocardial enzymes, electrolytes, amylase, coagulation parameters, B‐type natriuretic peptide (BNP), and procalcitonin—revealed no significant abnormalities. Electrocardiography indicated sinus tachycardia. Noncontrast CT scans of the head, chest, and abdomen showed only mild dependent atelectasis in the lower lobes bilaterally, without evidence of structural brain injury, pulmonary edema, or other organic pathology. Following admission, a multimodal detoxification protocol was rapidly implemented. Interventions included nasal cannula oxygenation, intravenous fluid therapy combined with forced diuresis to enhance systemic drug clearance, and empirical antimicrobial prophylaxis to prevent secondary infection. Electrolyte and acid‐base homeostasis were closely monitored and corrected as needed. Abdominal CT revealed persistent high‐density opacities within the gastrointestinal lumen—morphologically consistent with incompletely dissolved tablets—providing objective radiographic evidence of ongoing gastrointestinal drug absorption. Consequently, early gastrointestinal decontamination was initiated, comprising oral 20% mannitol (250 mL), single‐dose activated charcoal (30 g), and whole‐bowel irrigation with polyethylene glycol electrolyte solution, all intended to accelerate fecal elimination of unabsorbed drug. To remove absorbed toxins from systemic circulation, a structured “211” hemoperfusion regimen was instituted: two 3 h sessions on Day 1 (with a 12‐h interval), followed by one session each on Days 2 and 3, using the Jianfan HA330 resin‐based hemoperfusion cartridge. Blood and urine samples were collected prior to the first hemoperfusion session for toxicological screening. Samples not analyzed within 24 h were stored at 4°C; those delayed beyond this period were transferred to −20°C or lower for long‐term preservation.

Approximately 4 h after admission, the patient experienced acute respiratory decompensation, with peripheral oxygen saturation declining to 75%. Arterial blood gas analysis confirmed hypoxemia, revealing an oxygenation index (PaO_2_/FiO_2_) of 145 mmHg, consistent with acute respiratory failure. Bedside chest radiography showed no signs of pulmonary edema, pneumothorax, or pleural effusion. Emergency endotracheal intubation and mechanical ventilation were performed, resulting in rapid improvement in oxygenation, with SpO_2_ restored to 95%–100%. Over the subsequent hours, the patient developed transient facial twitching and upper limb myoclonus, generalized tremors, hyperthermia (peak temperature: 39.5°C), and persistent tachycardia (heart rate: 120–130 beats/min). These manifestations prompted initiation of physical cooling and antipyretic pharmacotherapy. Concurrently, blood, urine, and sputum cultures were obtained, along with inflammatory markers including C‐reactive protein (CRP), procalcitonin, and complete blood count. Follow‐up results showed no elevation in inflammatory indices, and all microbiological cultures remained negative. Repeat chest CT demonstrated no new consolidative, ground glass, nodular, or exudative pulmonary changes. Given the temporal association with polypharmacy involving serotonergic agents, the characteristic progression of neuromuscular and autonomic symptoms, and the exclusion of infectious, metabolic, and structural neurological etiologies—including NMS and anticholinergic toxicity, it was confirmed that the case was SS related to an overdose of quetiapine fumarate. Subsequent management emphasized intensified toxin removal through continued hemoperfusion, maintenance of mechanical ventilation, and administration of dexmedetomidine for sedation to mitigate autonomic instability.

On hospital Day 3, following successful weaning from sedation, the patient regained full consciousness, obeyed commands appropriately, and maintained a maximum temperature of 38.0°C, allowing for safe extubation. Toxicological analyses of blood and urine samples collected after completion of four hemoperfusion sessions were completed (see Table 1). By Day 4, peak body temperature had normalized to 37.3°C, and vital signs remained stable. However, persistent emotional lability and behavioral disturbances warranted psychiatric evaluation, leading to transfer to a specialized mental health facility for ongoing care.

**Table 1:** Serum and urine levels of psychotropic agents before first and after fourth hemoperfusion (Day 1 and Day 3).

Group		Alprazolam concentration (ng/ml)	Lorazepam concentration (ng/ml)	Quetiapine concentration (ng/ml)
Serum	D1	65.7	333	1449
	D3	8.0	35.2	129
Urine	D1	110	138	1376
	D3	4.2	21.9	54.2

## 3. Discussion

SS is a potentially life‐threatening condition resulting from excessive serotonergic activity in the central nervous system due to elevated synaptic serotonin (5‐HT) levels [5]. It typically arises acutely following overdose or concomitant use of drugs that enhance 5‐HT neurotransmission. Approximately 60% of patients manifest symptoms within 6 h, and in severe cases, the disease progresses rapidly. Without prompt recognition and intervention, SS may swiftly deteriorate into multiorgan failure or death. Common causative agents include selective serotonin reuptake inhibitors (SSRIs), MAOIs, and serotonin–norepinephrine reuptake inhibitors (SNRIs) [6]. Notably, several nonpsychiatric medications can also precipitate SS through pharmacokinetic or pharmacodynamic interactions, including analgesics (tramadol, fentanyl, dezocine, and oxycodone) [7], triptans for migraine, the antibiotic linezolid [8], the antiviral nirmatrelvir/ritonavir (Paxlovid) [9], and methylene blue, a redox dye with MAO‐inhibiting properties [10]. Quetiapine is mainly metabolized by CYP3A4. Inhibition of CYP3A4 activity can significantly increase the blood concentration of quetiapine, which may lead to toxic symptoms, especially when multiple CYP3A4 inhibitors are ingested. Common CYP3A4 inhibitors include ketoconazole, itraconazole, clarithromycin, ritonavir, nefazodone, and grapefruit juice, and so on [11]. These findings underscore the importance of heightened awareness among clinicians—particularly physicians and pharmacists—regarding polypharmacy risks to prevent serious adverse outcomes.

We applied the Naranjo adverse drug reaction probability scale to assess the causality of the quetiapine fumarate–associated adverse reaction. A total score of 8 was obtained, corresponding to the “probable” category—indicating a high likelihood of a causal relationship. The itemized scoring and rationale are detailed in Table 2. Diagnosis of SS primarily relies on the Hunter Serotonin Toxicity Criteria [12], which require a history of 5‐HT–enhancing drug use within the past 5 weeks and the presence of at least one of the following: (1) tremor and hyperreflexia; (2) clonus (spontaneous, inducible, or ocular); (3) muscle rigidity with temperature > 38°C plus clonus or nystagmus; (4) nystagmus with irritability or diaphoresis; or (5) clonus with irritability or diaphoresis. The patient in this report presented after intentional ingestion of alprazolam, lorazepam, and quetiapine fumarate. Within 8 h of admission, the patient developed hyperthermia (peak temperature 39.5°C), upper limb myoclonus, generalized tremor, and agitation—fulfilling Criterion 3 and 5 of the Hunter Serotonin Toxicity Criteria (clonus plus agitation). However, quetiapine exhibits dual pharmacological activity—antagonism at dopamine D2 receptors and partial agonism at serotonin 5‐HT1A receptors—rendering it theoretically capable of contributing to features of both SS and NMS. So we examined the complete clinical data of the patient and confirmed that the creatine kinase (CK) levels continuously monitored after admission were always within the normal reference range, and there was no severe muscle rigidity or typical “lead‐pipe rigidity”. According to the Levenson diagnostic criteria [13], the diagnosis of NMS requires the presence of all three major criteria (fever, muscle rigidity, and elevated CK) or two major criteria plus at least four minor criteria (tachycardia, abnormal blood pressure, altered consciousness, hyperhidrosis, and leukocytosis). This case did not meet the above Levenson diagnostic criteria. Therefore, the final diagnosis of this case was SS rather than NMS.

**Table 2:** Naranjo algorithm.

Number	Evaluation issues	YSE	NO	Unknown
1	Have you ever had a similar reaction to this drug in the past?	+1	0	0√
2	Did the adverse event occur within a reasonable time sequence?	+2√	−1	0
3	Did the symptoms improve after stopping or reducing the dosage of the medication?	+1√	0	0
4	Did the symptoms recur after taking the medicine again?	+2	−1	0√
5	Are there any other possible explanations?	−1	+2√	0
6	Does a placebo induce the same reaction?	−1	+1√	0
7	Has the blood drug concentration reached a toxic level?	+1√	0	0
8	Is the dose‐response relationship clear?	+1√	0	0
9	Have the response patterns of previous patients to this drug been consistent?	+1	0	0√
10	Have the response patterns of previous patients to this drug been consistent?	+1	0√	0

*Note:* The total score is the sum of all individual scores, with a range from −4 to +13 points: ≥ 9 Definite, 5–8 probable, 1–4 possible, ≤ 0 doubtful.

Alprazolam and lorazepam are benzodiazepine sedative‐hypnotics commonly encountered in acute poisoning, typically presenting with altered mental status and respiratory depression—features that usually prompt immediate clinical attention. Quetiapine fumarate, a second‐generation antipsychotic, exerts its effects primarily through antagonism of dopamine D2 and serotonin 5‐HT2A receptors, contributing to antipsychotic and mood‐stabilizing properties. Its active metabolite, norquetiapine, exhibits comparable D2 affinity but greater 5‐HT2A receptor blockade, potentially amplifying serotonergic effects. Literature reports indicate that ingestion of quetiapine fumarate tablets exceeding 3 g or a serum quetiapine concentration above 2,000 ng/mL is associated with a significantly elevated risk of severe clinical manifestations, including GCS score ≤ 8, requirement for endotracheal intubation, hypotension, QTc interval prolongation, and seizures [14]. Moreover, coingestion with benzodiazepines markedly potentiates toxicity: such severe outcomes may occur even at lower quetiapine doses or serum concentrations [15]. Globally, quetiapine use has risen substantially. A 2022 systematic review reported that Australian prescriptions reached 1 million in 2015—a 285‐fold increase since its market introduction in 2000. The 2018 American Association of Poison Control Centers′ annual report ranked antipsychotics as the second most common class involved in adult drug exposures, surpassed only by analgesics, with quetiapine frequently implicated due to expanded off‐label use and misuse. In contrast, reports of acute quetiapine poisoning in China remain limited, predominantly published as case reports [16]. Evidence indicates that quetiapine overdose can lead to severe complications, including acute respiratory distress syndrome (ARDS) [17], NMS [18], and SS—particularly when coadministered with other serotonergic agents [19]. In this case, holiday‐related delays precluded early blood and urine toxicology screening, preventing quantification of individual drug concentrations. Thus, diagnosis was inferred from the temporal evolution of clinical features. Initial presentation with coma and hypoxemia aligned with benzodiazepine toxicity; however, persistent tachycardia and lack of miosis deviated from typical sedative overdose patterns, suggesting additional pathophysiological mechanisms. Shortly after admission, the patient developed hyperthermia and myoclonus, raising suspicion for sepsis. Repeat testing showed normal CRP and procalcitonin levels, negative cultures from blood, urine, and sputum, and no radiological evidence of infection, effectively ruling out infectious etiologies. Integrating the drug ingestion history, known pharmacology of quetiapine, and clinical trajectory, SS that might eventually be related to quetiapine. The mechanism underlying quetiapine‐associated SS remains incompletely elucidated but is increasingly understood as indirect and multifactorial. Clinical evidence indicates that quetiapine—primarily via potent 5‐HT₂ₐ receptor antagonism—disinhibits serotonergic neurotransmission, thereby augmenting synaptic 5‐HT₁ₐ receptor stimulation indirectly, particularly in the presence of coadministered serotonergic agents [20]. At supratherapeutic doses, enhanced 5‐HT₂ₐ blockade further amplifies this disinhibition, increasing synaptic availability of both serotonin and dopamine; the resultant net increase in 5‐HT₁ₐ activation contributes to the characteristic hyperadrenergic and neuromuscular features of SS [21]. Critically, norquetiapine‐the major active metabolite of quetiapine—exerts direct, high‐affinity partial agonism at 5‐HT₁ₐ receptors, providing a complementary, dose‐dependent pharmacodynamic pathway that synergizes with 5‐HT₂ₐ‐mediated disinhibition to elevate SS risk [22]. With aggressive supportive care and enhanced elimination via hemoperfusion, the patient′s condition improved progressively and he was discharged without sequelae.

Management of SS hinges on early recognition, immediate discontinuation of offending agents, and robust supportive therapy. According to the extracorporeal treatments in poisoning (EXTRIP) workgroup, hemoperfusion is recommended for toxins with high volume of distribution, significant lipid solubility, and high protein binding‐characteristics that align closely with quetiapine (lipid‐soluble, ~83% plasma protein bound). Although hemodialysis has limited efficacy for such compounds, it may support fluid and electrolyte homeostasis in critically ill patients. Combining extracorporeal modalities may offer synergistic benefits in severe poisoning [23]. Mild cases generally resolve within days with supportive measures alone, whereas severe cases demand intensive interventions: benzodiazepines or dexmedetomidine for agitation and autonomic instability, intubation if needed for airway protection, and active cooling to mitigate metabolic demand. Adjunctive use of cyproheptadine, a 5‐HT2A antagonist, is supported in severe SS [24]. A retrospective analysis by Prakash et al. of 56 fatal SS cases found that 50% died from cardiac complications, and 84% had not received cyproheptadine—highlighting a potential therapeutic gap and reinforcing the need for early administration in critical cases [25].

Beyond single‐drug toxicity, quetiapine is more commonly involved in polysubstance overdoses, particularly with antidepressants, benzodiazepines, alcohol, acetaminophen, or antiepileptics. Extended‐release formulations pose higher risks due to prolonged release and cumulative exposure, especially when combined with CNS depressants. Coingestions often mask classic symptoms, leading to diagnostic delays. Early toxicology screening is therefore essential to identify dominant toxins and guide targeted management. The successful outcome in this case stemmed from timely recognition of atypical SS features, accurate differentiation from sepsis, and avoidance of unnecessary antibiotics and prolonged mechanical ventilation. This underscores the necessity for emergency clinicians to maintain a high index of suspicion for rare but life‐threatening drug interactions, particularly in complex poisoning scenarios.

However, a limitation of our toxicokinetic assessment is that drug concentrations were measured only after completion of multiple hemoperfusion sessions—thus precluding reliable estimation of peak exposure and meaningful dose–response correlation. Although serial measurements before and after each hemoperfusion session would have strengthened pharmacokinetic interpretation, this was not feasible due to financial constraints: The family declined further testing owing to inability to bear the associated costs.

## 4. Conclusion

The therapeutic effect of quetiapine fumarate tablets is primarily attributed to its antagonism of central dopamine D2 and 5‐hydroxytryptamine (5‐HT2A) receptors. However, in cases of drug overdose, it may induce clinical manifestations such as altered mental status, motor dysfunction, and autonomic instability—symptoms that meet the diagnostic criteria for SS. Despite this, SS is frequently misdiagnosed or overlooked in clinical practice, particularly in the context of polypharmacy or combined drug intoxication. Given that delayed diagnosis and inadequate intervention are associated with substantial morbidity and mortality, early recognition of characteristic clinical features and the prompt initiation of targeted treatment are critical for improving patient outcomes and enhancing the likelihood of successful recovery.

## Funding

No funding was received for this manuscript.

## Ethics Statement

The medical records and biological specimens used in this study were retrospectively obtained from routine clinical care. Accordingly, we sought and received formal approval from our institutional ethics committee for waiver of informed consent.

## Consent

All the patients allowed personal data processing and informed consent was obtained from all individual participants included in the study.

## Conflicts of Interest

The authors declare no conflicts of interest.

## Data Availability

The data that support the findings of this study are available from the corresponding author upon reasonable request.
